# Epidemic Influenza

**Published:** 1890-09

**Authors:** Wm. Johnstone Fyffe


WM. JOHNSTONE FYFFE, M.D., *President*.

---

I AM not aware of any reason why I should have been asked to open a discussion to-night on the subject of Influenza, except this one: that as Physician to Clifton College I have had an opportunity, during the months of January, February, and March, of witnessing an outbreak of this disease among the members of the School—an outbreak which has affected more or less all classes of persons in the School-community, masters, boys, officials, and servants.

It is not my intention to occupy your time in discussing the history of this remarkable malady. The literature on the subject is no doubt familiar to you all.

As my remarks are only intended to be introductory to the discussion which will follow, I shall confine myself to a brief account of the recent epidemic as it came directly under my personal notice.

I shall state how and where it began in the School, and the manner of its spread and distribution. I shall put before you the total number of cases which have occurred in the College and in each Master's House separately. I shall then speak of the clinical features of the disease—its symptoms, duration, complications, and sequelæ; and I shall finish my paper with a few general propositions which may perhaps furnish some points for your consideration and discussion this evening.

The following table will indicate at a glance the parts of the School in which the epidemic appeared, either lightly, severely, or not at all.

*Table showing Percentage of Cases of Influenza in the Masters' Houses in Clifton College.*

THE COLLEGE—NINE HOUSES.

| | No. of Boarders. | Cases of Influenza. | Percentage. |
|---|---|---|---|
| A. | 11 | 5 | 45.4 |
| B. | 15 | 4 | 26.6 |
| C. | 48 | 5 | 10.41 |
| D. | 48 | 7 | 14.50 |
| E. | 48 | 20 | 41.66 |
| F. | 48 | 8 | 16.66 |
| G. | 48 | 10 | 20.85 |
| H. | 10 | 1 | 10.00 |
| I. | 73 | 28 | 38.35 |
| Total | 349 | 88 | 25.41 |

JUNIOR SCHOOL.

| | No. of Boarders. | Cases of Influenza. | Percentage. |
|---|---|---|---|
| A. | 40 | 1 | 2.50 |
| B. | 25 | 3 | 12.00 |
| C. | 8 | 0 | 0.0 |
| Total | 73 | 4 | 5.30 |

PREPARATORY SCHOOL.

| | | | |
|---|---|---|---|
| A. | 13 | 6 | 46.15 |

SUMMARY.

| | | | |
|---|---|---|---|
| College | 349 | 88 | 25.41 |
| Junior School | 73 | 4 | 5.30 |
| Preparatory | 13 | 6 | 46.15 |
| Total | 435 | 98 | 22.83 |

TOWN BOYS.

| | No. of Boys. | Cases of Influenza. | Percentage. |
|---|---|---|---|
| College | 137 | 62 (College, Junior School and Preparatory together) | |
| Junior School | 64 | | |
| Preparatory | 33 | | |
| Total | 234 | 62 | 26.40 |

| *Total Strength of the School.* | *Total No. of Cases.* | *Percentage.* |
|---|---|---|
| 669 | 160 | 23.9 |

The first cases of influenza occurred on the 27th January, just a week after the opening of the School Term, in the Preparatory School and in letter I House. Two cases were admitted to the Sanatorium from the former, and four from the latter—six cases in all,—on the first day of the epidemic.

The progress of the disease from this time was rapid, and in the third week of the Term it had reached its height. You will observe from the table some peculiarities about it. You will remark the extraordinary immunity from it which the Junior School enjoyed in comparison with the Upper School, or College proper: only four cases occurred among the boarders in the Junior School out of a strength of 73; and in the largest Junior House, containing 40 boys, there was only one case. You will also observe that in the Upper School the main weight of the epidemic fell upon three Houses marked in the table E, I, and A; and it also severely affected the little boys in the Preparatory School, whose ages are from 8 to 10.

In the House marked E 19 cases occurred within a few days, and four servants were attacked; and I felt it to be my duty to advise the master of this House to give the parents of the boys the option of removing them to their homes for a fortnight, if they thought it desirable. About ten boys went home. The remainder stood their ground.

You may naturally ask, Were there any local circumstances to account for the rapid spread of the disease in these three Houses: were they in any sense in an unsanitary state? I think I can give a decided negative to the question. Every boarding-house is examined once every Term by a competent sanitary inspector, acting under my orders. I think I can safely affirm that every House is in a good sanitary condition, while some are models of good sanitation. While I cannot charge the prevalence of the epidemic in these four Houses to any special sanitary fault, I think there is an undoubted tendency in the disease to attack with great rapidity persons who are congregated in numbers together.

With regard to the clinical features of influenza, I have divided the cases which have come under my notice into three classes: (1) Mild uncomplicated cases; (2) Severe uncomplicated cases; and (3) Cases complicated with lesion of some organ.

Out of the entire number of 98 cases, there were six severe uncomplicated cases, and eight complicated with dangerous lung-lesion.

There was a great similarity in the onset of the symptoms in the mild cases. The boys were attacked as a rule suddenly, either when at work in school or after games of football or fives. Sometimes the attack came on early in the morning, sometimes late at night. The premonitory symptoms even in severe cases appeared to be of very short duration, the first symptom being generally chilliness or giddiness. In one of the severest cases the boy had been playing hockey on the Down in the afternoon, came home, changed his clothes, felt apparently well at six o'clock, joined his school-fellows at tea, and made a hearty meal. I was sent for at eight. He had been seized with a rigor, and severe symptoms of influenza had developed in that interval. In the mild cases, the symptoms when developed were generally headache, pains in the loins, sides, and thighs, pyrexia, and great prostration.

In all my cases, whether mild or severe, catarrhal symptoms were conspicuous by their absence. I observed red and suffused eyes occasionally, but coughs and general symptoms of catarrh I seldom observed. In one case there was pharyngitis.

In the mild uncomplicated cases the temperature seldom exceeded 102°. But headache, principally frontal, and pains in the chest, back, and thighs, were universally present, together with an extraordinary amount of prostration and weakness—a condition entirely out of proportion either to the severity of the fever or its duration, but indicative of a profound and sudden impression having been made on the nervous system. This was well exemplified in the case of a boy named B——. In this case the fever only lasted two days, the temperature never reached 100°, and was subnormal on the third day; and yet the boy, otherwise a healthy lad of 17, was so prostrated he could not stand or walk for nearly a week; and although his case was entirely uncomplicated, he did not recover his strength sufficiently to enable him to resume his school-work, and I was obliged to send him home for the remainder of the Term.

In the six severe uncomplicated cases, I could discover no lesion of any organ. The symptoms were those of a fever pure and simple; but a fever acute and severe, lasting from four to six days, ushered in by chilliness and rigor, accompanied by headache of a very severe character, and the usual pains about the trunk and thighs, but in an intensified form. There was great restlessness—in some cases insomnia, and a general feeling of misery and discomfort.

In some of these instances I observed severe pain in the eyeballs, which were tender and tense. In most of them there was very little disturbance of the digestive organs. Constipation was occasionally present; but as a rule the tongue was unusually clean.

Certainly the most prominent feature was great depression and debility. Fortunately, vomiting was seldom present, so that food and stimulants could be freely administered. As I have said, the duration of the fever in these cases was from three to seven days. The defervescence when it occurred was sudden and sharply defined, the temperature falling at once to the normal point, or nearly so, often becoming subnormal and remaining so for several days. With the fall of temperature, the rapid pulse, headache, and pains disappeared, but leaving still to be combated the sequelæ of which I shall presently speak.

In these cases the highest temperature recorded was 105.4°; but the curve generally was from a maximum of 104° to 101° for five or six days, and then a drop to the normal point or below it.

I think these severe uncomplicated cases exemplify the hypothesis that influenza is a specific fever depending upon a blood-poison.

When the thermometer indicates a body heat of 105 degrees or more, we are usually on the alert to discover some organ affected with inflammation. But in these cases there was nothing of the kind. As soon as the fever ceased, the patient was relieved and no lesion of any organ could be discovered. The poison, whatever its nature may be, had passed through its life-history, had been planted, had developed, culminated, and died, leaving, it is true, behind it unmistakable evidences of its power, but no permanent damage.

I now come to speak of the third class of cases which have come under my notice—cases of influenza, complicated with inflammation of some organ, either the lungs, bronchial membrane, pleuræ, or with an inflammatory condition of the bowels, or with lesion of the cerebro-spinal system.

I believe I am right in stating that in by far the majority of cases in which complications have arisen in the recent epidemic, the lungs have been the principal organs affected. Either pneumonia, pleurisy, or bronchitis has in a certain number developed early in the course of an attack of influenza, and from one or other of these causes that mortality has taken place which, during the months of December, 1889, and January, February, and

March, 1890, has produced a large increase in the death-rate in many of the cities and towns of the kingdom.

I think there is some risk during the prevalence of epidemic influenza, especially during the existence of cold weather, of confounding cases of simple pneumonia which might have occurred without the presence of influenza at all with cases which owe their origin distinctly to the epidemic.

But in addition to the fact that there are generally distinct symptoms of influenza before the development of pulmonary mischief, the distinguishing feature of the inflammation which complicates it is the sudden violent and rapid progress which characterises the inflammation, and the formidable symptoms which accompany it.

Whether we have to deal with bronchitis or pleurisy, or with broncho- or croupous pneumonia, we are confronted with a train of symptoms of a very aggravated character. We have, in fact, pulmonary inflammation, serious enough in itself, but intensified by the presence of a specific fever. In other words, we have pneumonia or bronchitis or pleurisy *plus* influenza. Life is placed in the greatest peril in the course of a few hours, and the tendency to death from extreme prostration and cardiac failure requires the most incessant vigilance on the part of the physician and nurses.

This is well illustrated by the following case: C. J., a healthy schoolboy aged 16, was attacked with influenza on the 10th March. The symptoms were not very severe at first: he had headache, pains in the limbs and back, and his temperature was not above 101°. On the morning of the 12th he ate a good breakfast, and was in good spirits. At 11 a.m. he was seized with a rigor, his temperature rose to 105.3°, respiration became rapid, and pulse 112, with a marked intermission. The same evening the usual signs of broncho-pneumonia were found in the base of the right lung. The disease rapidly increased, and ran its course with great gravity and severity; respiration rose to 40 in the minute, there was great restlessness, severe pains in the side, insomnia, and loud and incoherent delirium. These symptoms lasted for six days; the pulse was extremely weak and intermittent, and the tendency to death from cardiac failure placed this boy's life in imminent peril. But sudden and rapid defervescence took place on the seventh day. In this case the physical signs were more those of broncho- than of croupous pneumonia. There was a great deal of coarse crepitation at the base of the right lung, and an immense quantity of thick adhesive rusty sputum was coughed up.

This case was complicated also with acute abdominal tympanites, a symptom which gravely increased the severe dyspnœa. At the suggestion of my friend, Dr. E. L. Fox, turpentine was administered, with the best possible results. Recovery was rapid and complete, and there were no sequelæ.

The next case, that of W., was the worst, and terminated fatally. The illness began on the 1st of March with influenza; but on the 3rd the most intense form of croupous pneumonia set in, embracing nearly the whole of the left lung. From this time the symptoms were most formidable. Although the temperature was not so high as in other cases, only once having reached 104°, yet the other symptoms were so severe as to give one the impression of profound septic poisoning:—wild incoherent delirium, extreme prostration, a dusky aspect, insomnia, respiration over 50 in the minute, and pulse 120 to 130; urine, slightly albuminous. The symptoms were in fact of a malignant character, and nothing in all probability would have prevented a fatal issue.

The case of W. B. is another example of pneumonia complicating influenza. The symptoms of inflammation of the right lung set in on the third day. The temperature suddenly rose to 106.2°. There was violent vomiting, continued for several hours, and very great prostration; but there was no delirium. The case ran its course satisfactorily; but convalescence was retarded by severe and prolonged debility, coupled with anæmia.

I do not think it necessary to give you details of all the cases of pneumonia which occurred during the epidemic, as there was much similarity between them; but I may mention the case of R. W., a lad of 17, exceedingly tall for his age, measuring 6 feet 4 inches. He was attacked with influenza on the 4th February. The symptoms were very severe from the first. This was a second attack: he had had the disease in a mild form during the Christmas holidays. There was very severe headache, and the pains in the chest, sides, and legs were of the most intense character. There was profuse sweating, restlessness, and some delirium, but not much. About the fourth day there were physical signs of inflammation in the left side of the chest, which was diagnosed as pleuro-pneumonia; but in observing the progress of the case it became evident that the left pleura was principally engaged. Fluid began to collect in the left side to a considerable extent, displacing the heart. After treatment for four weeks, it became pretty evident that the case was one of empyema. An exploratory puncture was made; and having ascertained that the contained fluid was purulent, an incision was made in the chest by Mr. Harsant, and about a pint of pus removed. A drainage-tube was inserted, and the case was treated by Mr. Harsant with the usual antiseptic precautions. The result has been satisfactory. Immediately after the operation the symptoms all began to improve. The temperature fell to the normal point and appetite returned. The wound has now completely healed. The patient is now quite convalescent and has gone to the seaside. Some damage was left on the affected side; but on the last occasion when I examined him great improvement had taken place. Respiratory sounds were heard fairly well at the base and apex of the left lung, although rather feeble in the middle part on which the weight of the inflammation fell.

I have two other cases which were under my care (not among the College boys) which I would like to mention. I was called to see a lady, aged 30, married, one morning towards the end of February. I found her with the usual symptoms of influenza; but being a delicate and rather anæmic woman, her prostration and weakness were very great. I thought it right to see her again at mid-day. I then found her suffering from violent diarrhœa: I think she had thirteen actions from the bowels during the day. Just as I was going to bed that night at eleven o'clock, her husband came to me in great alarm to say his wife was bleeding profusely from the bowels. I went at once to the house, and found that she had passed about half-a-pint of blood *per anum*. I placed her upon frequent doses of hazeline. There was no further hæmorrhage, and the diarrhœa ceased next day. There was very severe pain and tenderness over the cæcum. These symptoms were followed by such severe prostration, that I felt considerable alarm for her safety. This, I presume, was an instance of the gastro-intestinal form of influenza.

One of the matrons in one of the College boarding-houses, a woman aged 42, was attacked with influenza on the 15th March. She had headache, feverishness, and some bronchial *râles* over the chest. In three days she was much better, got up, and attempted to perform some of her duties; but she was suddenly seized with loss of motor power in the lower extremities: she could not stand, and she was unable to move her legs off the bed. There was also loss of motor power in the arms, but not so marked as in the legs. There did not appear to be any loss of sensation. The knee-jerk and plantar reflex were entirely absent. There was constant tingling in the soles of the feet and in the palms of the hands; there was also tingling in the lips, on the dorsum of the tongue and the left side of the palate; there was some difficulty in deglutition; there was left facial paralysis, but the orbicularis palpebrarum muscle was unaffected; the mouth was drawn to the right side, and the left eyelid did not close completely; food collected in the left side of the mouth; there was no giddiness and no definite sense of constriction round the waist.

These serious symptoms lasted for, I think, five weeks; but gradually and slowly passed off, and the patient has completely recovered in every respect, except that the knee-jerk has not yet returned.*

I may here mention a case, the notes of which have been furnished to me by my son, who is Resident Physician of St. George's Hospital. It was the case of a distinguished student of the Hospital, who, after an attack of influenza, developed paraplegia, and died after a week's illness. The case was considered to be one of myelitis; but at the *post mortem* examination no disease of brain or

* The question is, Was this a case of acute ascending Landry's paralysis?

spinal cord was discovered. There was a great quantity of muco-pus in the medium and smaller bronchial tubes, and an immense quantity of petechial spots scattered over the surface of both lungs.

Much has been written about the cutaneous rash which is said frequently to accompany influenza, and which gave rise to the suspicion at one time that the epidemic was one of Dengue or dandy fever. I have only seen a rash in one instance, and then it was not very well defined. It appeared as an erythematous evanescent rash on the face in one case on the second day of the attack. In very young patients I believe it is occasionally seen. My son, who has seen 360 cases during the epidemic, tells me he has only seen the rash in young children. I have seen an eruption of herpes labialis in several cases, even in the mild ones.

With regard to the sequelæ of influenza, the most prominent and most fatal have been from pulmonary inflammation. For a considerable time after even a mild attack of influenza patients are liable to attacks of pneumonia, bronchitis, or pleurisy, and from this cause mainly the mortality has arisen. Probably this may have been owing, in many instances, to imprudence on the part of patients in going out too soon after an attack during the prevalence of cold weather. But undoubtedly there is a marked liability to pulmonary inflammation after an attack of influenza has passed off and the patient is apparently convalescent.

Then another very important and troublesome consequence of it is, the persistent weakness and debility which results from an attack. Even in the young, strong, and healthy subjects, with whom I have had principally to deal, this feature was most prominent. What is the nature of this asthenia? It seems to me that it arises mainly from cardiac weakness; and I observe that Dr. Wilks has drawn attention to this fact in a recent number of the *Lancet*, giving four instances where death ensued from heart-failure after recovery from influenza, without the presence of any organic lesion whatever. The fact is, that from the beginning to the end of an attack of influenza, throughout convalescence, and for many weeks after, the pulse is poor, weak, and often intermittent, and the temperature often subnormal.

Then another of the troublesome sequelæ of this disease is mental depression, lowness of spirits, an indifference to the usual interests of life which is difficult to shake off: and I know at least of one instance in which melancholia followed upon an attack of influenza; and Dr. Bertillon, chief of the Statistical Department of Paris, states that suicides increased 40 per cent. during the prevalence of the epidemic.

Anæmia, or rather spanæmia, is another consequence which I have distinctly met with; and I need not remind any who have been dealing with the epidemic of the many forms of neuralgia which have presented themselves—frontal, temporal, facial, and especially post-orbital—the last, in my experience, having been the most severe and persistent.

I can well understand how serious must be the results of this disease in those cases where there has been previous chronic disease of the lungs, such as bronchitis or phthisis. But such cases have not come within the range of my recent experience, and I must leave others to speak of them.

Now as to Treatment. The mild cases required hardly any medicinal treatment. I followed the fashion frequently, and gave antipyrin where the headache was severe or the temperature high—5, 8, or 10 grains, according to the age of the patient, every two hours. I believe it relieved the headache and reduced the fever a little; but I never pushed it far. I avoided salicylate of soda religiously: there was enough cardiac depression without it. As soon as the fever abated, and indeed often before that, I gave quinine and placed the patient on a generous diet, with champagne or burgundy. In the severe and complicated cases, the whole treatment was directed to avert the tendency to death from cardiac failure. Stimulants were freely administered with food, and ammonia, bark, ether, and digitalis were given according to circumstances. But what I relied on most was the assiduous and devoted care and attention of an excellent matron and a staff of admirable nurses, supplied to me by the Royal Infirmary and General Hospital.

For the relief of the severe neuro-muscular pains in the chest, sides, and thighs, I used only one remedy—a turpentine fomentation—and found it unfailing; it always gave immediate relief.

I have now put before you—very imperfectly, I am afraid—an unvarnished account of my own personal experience in the recent epidemic. You will observe it is an experience limited principally to one class—to healthy schoolboys. With reference to the effect of influenza on other sections of the community—on those more advanced in life, on the aged and infirm—I hope other members will favour us with the results of their experience.

Let me now, in conclusion, draw your attention to one or two propositions, which may possibly serve as points of interest to discuss—not that I have any wish to limit the discussion to these alone, but because they seem to me to have a somewhat practical bearing on the subject, and by gathering the collective experience of our members on them we may possibly learn something from the past which may help to guide under similar circumstances in the future.

I. What is the nature of influenza? Is it a specific fever? or is it merely catarrh in an epidemic form?

To that question I would reply, for my own part, that its natural history, so far as we know of it, and its clinical features, point to the hypothesis that it is distinctly a fever depending on a specific poison introduced, we do not know how, into the blood—a poison with a brief life-history, but producing sudden and often serious results on the nervous system and secondarily on various organs in the body.* I think that it is not merely an epidemic catarrh, as it appears that symptom has only existed in about 10 per cent. of the cases. But while I think the weight of evidence seems to prove its specific origin, the search for any distinctive germ or microbe pertaining to influenza has, so far as I have been able to ascertain, proved fruitless.

II. Whence is its local origin, and its mode of diffusion and distribution?

I am not going to attempt a solution of this question: I throw it out for the consideration of those who have made epidemiology their special study. It would seem that its starting-point is somewhere in Eastern Siberia; that it radiates in a direction from N.E. to S.W.; that it suddenly bursts like a thunderclap upon cities and crowded communities; that it travels with a rapidity far faster than

* What the nature of the poison may be—whether a germ or contagium vivum, or whether a miasm of telluric origin, malarial and air-borne—we cannot say.

is explicable by the conditions of human intercourse. We hear of it to-day in London, a few days later in New York, and after sweeping over the continent of the West it leaps back to India.

III. Is influenza in any sense a contagious or infectious fever?

I think it is a doubtful question. There are arguments *pro* and *con*. We see large numbers of persons who are thrown much together by the circumstances of their lives —postmen, employés in different places of business, soldiers in barracks, boys and girls in schools—struck down suddenly, almost too suddenly to admit of contagion being the cause. On the other hand, we find a large number of nurses and other attendants on the sick contracting it (there were over twenty nurses at one time ill with it in St. George's Hospital); and I certainly think it has a tendency to fix itself in houses, and to affect more or less every one of its susceptible inmates, either at once or in succession.

Lastly: What lessons can we learn from the recent epidemic? Can anything be done in future to stop or modify it? Is segregation of the sick possible or desirable? We take every precaution in the face of an epidemic of cholera —do we reflect that influenza is a disease which kills a larger number of people than cholera does, on account of its much more general distribution?* What is our duty with regard, for example, to communities over which we may exercise professional control?

Time will not permit me to say more, even if I wished to do so. I now leave the subject in your hands, not doubting that the discussion will lead to further elucidation of a disease the nature of which is still shrouded in much

* Graves's *Clinical Medicine*.

obscurity; which appears to be but little controlled by the laws which govern most epidemics; which is uninfluenced by sanitary precautions, or the neglect of them; which is independent of the vicissitudes of temperature, of climate, of prevailing winds, or the peculiarities of situation; which presents aspects of a perplexing character—attacking men in a much larger proportion than women and leaving young children comparatively free, spreading among poor and wealthy alike, suddenly overshadowing entire communities and continents in its progress, and leaving in its track very disastrous results.

